# Carbohydrate antigen 125 combined with N-terminal pro-B-type natriuretic peptide in the prediction of acute heart failure following ST-elevation myocardial infarction

**DOI:** 10.1097/MD.0000000000032129

**Published:** 2022-12-02

**Authors:** Kaizu Xu, Meifang Wu, Meinv Huang, Xiuping Zhuo, Yujuan Weng, Xi Chen

**Affiliations:** a Department of Cardiology, Affiliated Hospital of Putian University, Putian, China; b Department of Ultrasound, Affiliated Hospital of Putian University, Putian, China.

**Keywords:** B-type natriuretic peptide, carbohydrate antigen 125, heart failure, predictive value of tests, ST-elevation myocardial infarction

## Abstract

The value of serum carbohydrate antigen 125 (CA125) combined with N-terminal pro-B-type natriuretic peptide (NT-proBNP) in the evaluation of acute heart failure (AHF) after ST-segment elevation myocardial infarction (STEMI) remains unclear. The aim of this study was to evaluate the efficacy of CA125 combined with NT-proBNP in predicting AHF following STEMI. A total of 233 patients with STEMI were evaluated, including 39 patients with Killip II-IV and 194 patients with Killip I. The optimal cutoff point for predicting AHF was determined by receiver operating characteristic (ROC) curve, and the independent predictors of AHF were evaluated by multiple logistic regression. According to the cutoff value, it was divided into three groups: C1 = CA125 < 13.20 and NT-proBNP < 2300 (n = 138); C2 = CA125 ≥ 13.20 or NT-proBNP ≥ 2300 (n = 59); C3 = CA125 ≥ 13.20 and NT-proBNP ≥ 2300 (n = 36). Differences between groups were compared by odds ratio (OR). The levels of CA125 and NT-proBNP in AHF group were higher than those in non-AHF group (19.90 vs 10.00, *P* < .001; 2980.00 vs 1029.50, *P* < .001, respectively). The optimal cutoff values of CA125 and NT-proBNP for predicting AHF were 13.20 and 2300, both of which were independent predictors of AHF. The incidence of AHF during hospitalization was highest in C3 (69.44%), middle in C2 (20.34%) and lowest in C1 (1.45%). After adjustment for clinical confounding variables, compared with C1: C2 (OR = 6.41, 95% CI: 1.22–33.84, *P* = .029), C3 (OR = 19.27, 95% CI: 3.12–118.92, *P* = .001). Elevated CA125 and NT-proBNP are independent predictors of AHF in STEMI patients, and their combination can improve the recognition efficiency.

## 1. Introduction

ST-elevation myocardial infarction (STEMI) has been the focus of attention in cardiology community due to its high morbidity and mortality. In recent decades, the implementation of early reperfusion therapy and the advancement of adjuvant medications have contributed to the reduction of mortality in STEMI patients.^[1]^ Unfortunately, heart failure during hospitalization remains a common complication of acute myocardial infarction, with an incidence of 14% to 36%.^[2]^ There is currently reliable evidence that acute heart failure (AHF) after STEMI increases the risk of death during hospitalization and in the future.^[3–5]^ Therefore, how to simply and effectively predict the occurrence of early AHF after STEMI is an urgent problem to be explored in clinical practice.

N-terminal pro-B-type natriuretic peptide (NT-proBNP) is widely used in the diagnosis, treatment and prognosis assessment of acute or chronic heart failure, but it is easily affected by factors such as age and renal function.^[6]^ Serum carbohydrate antigen 125 (CA125), as a traditional tumor marker, has been shown to be valuable in guiding the treatment and evaluating the prognosis of AHF in recent years.^[7,8]^ Many studies have also confirmed the value of the combination of the aforesaid two biomarkers in the field of AHF,^[9,10]^ but there are few studies related to the application of STEMI complicated with AHF.

From the perspective of pathophysiology, cardiac insufficiency after STEMI is a mixture of congestion and inflammation.^[11]^ CA125, as a biomarker related to congestion and inflammation,^[12]^ and NT-proBNP, a peptide hormone released by mechanical stress stimulation of ventricular wall.^[6]^ The value of these two biomarkers with different internal mechanisms in the evaluation of AHF after STEMI is still unclear. The aim of this study was to analyze independent predictors of AHF, and to evaluate the efficacy of CA125 combined with NT-proBNP in predicting AHF following STEMI.

## 2. Methods

### 2.1. Study population

In this study, patients with STEMI who underwent primary percutaneous coronary intervention (PPCI) in the Affiliated Hospital of Putian University from June 2019 to June 2021 were selected as subjects, and the diagnosis of STEMI was based on the 2017 ESC STEMI Guidelines.^[13]^ Exclusion criteria: patients with non-occlusive coronary myocardial infarction, active infection, chronic heart failure, chronic lung disease, severe liver and kidney insufficiency, and malignant tumor. There were 246 eligible patients, including 7 patients who refused CA125 test, 4 patients who could not cooperate with hospitalized observation, 2 patients who died of sudden death after PPCI, a total of 13 patients were excluded, and 233 subjects were enrolled for final data analysis. All subjects were given informed consent and were willing to cooperate with standard treatment and be observed during hospitalization. The Ethics Committee of the Affiliated Hospital of Putian University approved the research protocol.

### 2.2. Research methods and data collection

After emergency coronary angiography, patients were treated strictly according to the guidelines. Coronary angiogram images were interpreted by the same senior specialist. Single vessel disease was defined as a narrowing of more than 50% of the main coronary artery (including left anterior descending coronary artery (LAD), left circumflex coronary artery (LCX), or right coronary artery (RCA) and/or their main branches. Multivessel disease were defined as > 50% coronary stenosis of more than 1 major coronary artery.^[14]^

Demographic data, past medical history, and physical examination were detailed in the cardiology department. Killip classifications system was applied to evaluate cardiac function after STEMI, defined as follows: Killip I, without HF and lung rales; Killip II, localized moist rales in the middle and lower lung (<50% lung field); Killip III, severe heart failure and pulmonary edema, moist rales throughout two lungs (>50% lung field); Killip IV, cardiogenic shock with different hemodynamic changes. Cardiac function grade ≥ Killip II was defined as AHF.^[15]^

Serum CA125 and NT-proBNP detection method: Fast-fasting venous blood was collected within 24 hours after admission, placed in a vacuum tube and stored in a low temperature refrigerator at −20°C. Serum CA125 level was determined by chemiluminescence immunoassay using CA125 II assay Roche Diagnostics (Model: MAGLUMI2000Plus; Test kit: Shenzhen Xinye Biomedical Engineering Co., Ltd., Shenzhen City, China). Serum NT-proBNP was quantitatively determined by dry immunofluorescence method using fluorescence quantitative analyzer (Model: YZB/SU 1124-2014; Test kit: Nanjing GP Based Egg Biotechnology Co., Ltd., Nanjing City, China).

### 2.3. Statistical analysis

All continuous variables were normality tested by Kolmogorov–Smirnov method. The continuous variables conforming to the normal distribution were represented by mean ± standard deviation ($\bar{x}\pm s$), and the independent-samples *t* test was used for comparison between groups. The continuous variables of non-normal distribution were represented by the median and the interquaternary interval (*Q1, Q3*), and the comparison between groups was performed by Mann–Whitney test. Categorical variables were expressed as frequency, and Pearson *χ*^*2*^ test or Fisher exact test was used for comparison between groups. Spearman’s rank correlation coefficient was used to evaluate the potential correlation between serum CA125 and NT-proBNP level and Killip classifications. Receiver operating characteristic (ROC) curve was used to analyze the optimal cutoff value of CA125 and NT-proBNP for predicting AHF after STEMI. Univariate and multivariate (forward method, *P* ≤ .10 into the equation, *P* > .10 remove the equation) logistic regression analyses were used to determine the independent predictors of AHF after STEMI. According to the cutoff value of CA125 and NT-proBNP (13.20 U/mL, 2300 pg/mL, respectively), all subjects were divided into 3 groups: C1 (CA125 < 13.20 U/mL and NT-proBNP < 2300 pg/mL, n = 138); C2 (CA125 ≥ 13.20 U/mL or NT-proBNP ≥ 2300 pg/mL, n = 59); C3 (CA125 ≥ 13.20 U/mL and NT-proBNP ≥ 2300 pg/mL, n = 36). Logistic regression model was used to analyze the odds ratios (OR) of AHF between each group of C1–C3 before and after adjusting for confounding clinical factors. MedCalc statistical software was used to draw area under curve (AUC) and do DeLong test. SPSS21.0 software (IBM Corp., Armonk, NY) was used for the remaining statistical analysis. *P* < .05 was considered statistically significant.

## 3. Results

### 3.1. Comparison of characteristics between patients with and without AHF

Compared with patients without AHF, patients with AHF had higher age, more females, higher on admission, higher proportion of previous hypertension and coronary heart disease, more renal insufficiency, and longer total ischemic time, more culprit vessel of LAD and disease, significantly higher CA125 and NT-proBNP, while proportion of RCA as a criminal culprit vessel was lower. There were no significant differences in heart rate, frequency of current smoker, diabetes, hypercholesterolemia, and culprit vessel of LCX between the two groups (Table 1).

**Table 1:** Baseline characteristics of patients with or without acute heart failure.

Items	With AHF (n = 39)	Without AHF (n = 194)	χ^2^, *z* or *t*	*P* value
Age (yr)	75.00 ± 5.70	62.54 ± 10.22	7.377	<.001
Sex/female [n (%)]	24 (61.54)	57 (29.38)	14.806	<.001
SBP (mm Hg)	133.77 ± 17.65	123.16 ± 21.41	2.901	.004
DBP (mm Hg)	84.62 ± 11.14	77.36 ± 15.85	2.725	.007
HR (beats/min)	78.62 ± 17.16	75.34 ± 18.30	1.032	.303
Current smoker [n (%)]	11 (28.21)	77 (39.69)	2.100	.147
Hypertension [n (%)]	25 (64.10)	77 (39.69)	7.862	.005
Diabetes [n (%)]	24 (61.54)	96 (49.48)	1.889	.169
Renal dysfunction [n (%)]	16 (41.03)	7 (3.61)	51.100	<.001
History of CHD [n (%)]	13 (33.33)	17 (8.76)	17.476	<.001
Dyslipidemia [n (%)]	15 (38.46)	72 (37.11)	0.025	.874
Total ischemic time (min)	479.00 (414.00,560.00)	320.00 (240.75,380.00)	−7.734	<.001
Culprit vessel [n (%)]				
LAD	33 (84.62)	68 (35.05)	39.517	<.001
LCX	3 (7.69)	40 (20.62)	3.605	.058
RCA	3 (7.69)	86 (44.33)	18.464	<.001
Multivessel disease	19 (48.72)	15 (7.73)	43.768	<.001
CA125 (U/mL)	19.90 (13.60–30.20)	10.00 (6.20, 12.80)	−7.661	<.001
NT-proBNP (pg/mL)	2980.00 (2222.00, 5388.00)	1029.50 (483.00, 2057.50)	−7.598	<.001

Continuous variables that satisfy normal distribution are expressed by mean and standard deviation, and continuous variables that do not satisfy normal distribution are expressed by median and quartile. Categorical variables are expressed by number and percentage.

AHF = acute heart failure, CA125 = carbohydrate antigen 125, CHD = coronary heart disease, DBP = diastolic blood pressure, HR = heart rate, LAD = left anterior descending branch, LCX = left circumflex branch, NT-proBNP = N-terminal pro-B-type natriuretic peptide, RCA = right coronary artery, SBP = systolic blood pressure.

### 3.2. Correlation and predictive efficacy of CA125 and NT-proBNP in AHF

Compared with Killip I group, CA125 and NT-proBNP in Killip II, III, IV group were significantly increased (all *P* < .05). Compared with Killip II group, CA125 and NT-proBNP in Killip III, IV group were significantly increased (all *P* < .05). Compared with Killip III group, CA125 and NT-proBNP were also higher in Killip IV group (*P* < .05).

**Figure 1.**
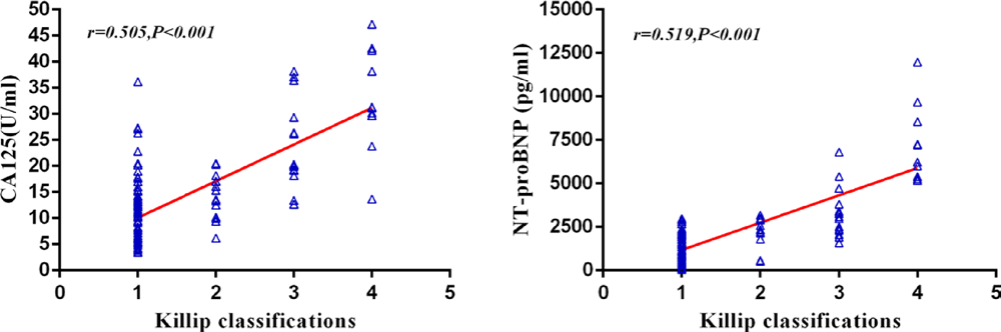
Correlation analysis of serum CA125 and NT-proBNP levels with killip classifications. CA125 = carbohydrate antigen 125, NT-proBNP = N-terminal pro-B-type natriuretic peptide.

**Figure 2.**
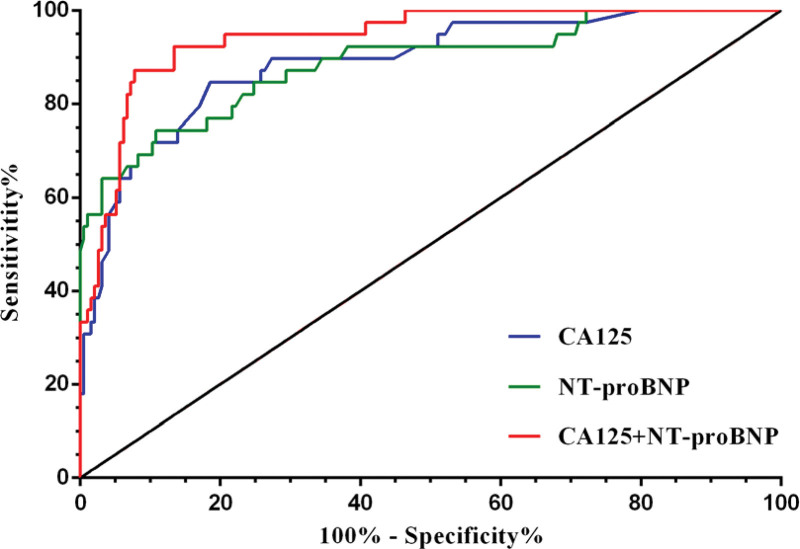
ROC curves comparing CA125, NT-proBNP and its combination for prediction of acute heart failure during hospitalization. CA125 = carbohydrate antigen 125, NT-proBNP = N-terminal pro-B-type natriuretic peptide, ROC = receiver operating characteristic.

Spearman’s rank correlation coefficient analysis revealed that CA125 increased gradually with the progress of Killip classifications (*R* = 0.505, *P* < .001. Meanwhile, NT-proBNP showed a similar trend (*R* = 0.519, *P* < .001) (Fig. 1). The ROC curves of CA125 and NT-proBNP on AHF were plotted (Fig. 2). It is found that the optimal cutoff value of CA125 was13.20 U/mL, and the AUC was 0.889, the sensitivity was 84.62% and the specificity was 81.44%. The optimal cutoff value of NT-proBNP was2300pg/ml, the AUC was 0.886, the sensitivity was 74.36%, and the specificity was 89.18%. When the two biomarkers were combined, the AUC was 0.941, the sensitivity was 87.18%, and the specificity was 92.27% (Table 2). The difference between ROC curves was compared by DeLong test based on AUCs, and it was found that the AUCs difference between CA125 and NT-proBNP was not statistically significant (difference between areas = 0.003, *P* = .935), while the AUC of CA125 + NT-proBNP was higher than that of CA125 (difference between areas = 0.052, *P* = .041) or NT-proBNP (difference between areas = 0.055, *P* = .022).

**Table 2:** Diagnostic value of serum CA125 and NT-proBNP in predicting acute heart failure.

Diagnostic indicator	AUC	Cutoff	Sensitivity (%)	Specificity (%)
CA125	0.889	13.20	84.62	81.44
NT-proBNP	0.886	2300	74.36	89.18
CA125 + NT-proBNP	0.941	–	87.18	92.27

AUC = area under curve, CA125 = carbohydrate antigen 125, NT-proBNP = N-terminal pro-B-type natriuretic peptide.

### 3.3. Multivariate logistic regression analysis of AHF following STEMI

Univariate logistic regression analysis showed that age, female sex, systolic blood pressure, DBP, history of hypertension or coronary heart disease, renal dysfunction, total ischemic time, culprit vessel of LAD, culprit vessel of RCA, multivessel disease, CA125 ≥ 13.20 U/mL, NT-proBNP ≥ 2300 pg/mL were the predictors of AHF after STEMI. Multivariate logistic regression analysis showed that CA125 ≥ 13.20 U/mL and NT-proBNP ≥ 2300 pg/mL were independent predictors of AHF (OR = 5.11, 95% CI: 1.56–16.75, *P *= .007 and OR = 3.26, 95% CI:1.07–9.96, *P* = .038, respectively). In addition, age (OR = 1.09, 95% CI: 1.01–1.17, *P* = .025), total ischemic time (OR = 1.01, 95% CI: 1.00–1.01, *P* = .015), culprit vessel of LAD (OR = 4.03, 95% CI: 1.21–13.44, *P* = .023) were also independent predictors of AHF following STEMI (Table 3).

**Table 3:** Logistic regression analysis of acute heart failure during hospitalization.

Items	Univariate analysis		Multivariate analysis	
Age	1.217 (1.139–1.300)	<.001	1.090 (1.011–1.174	.025
Sex/female	3.846 (1.881–7.863)	<.001		
SBP	1.024 (1.007–1.042)	.005		
DBP	1.033 (1.008–1.058)	.008		
HR	1.010 (0.991–1.028)	.303		
Current smoker	0.597 (0.281–1.269)	.180		
Hypertension	2.713 (1.328–5.545)	.006		
Diabetes	1.633 (0.808–3.302)	.172		
Renal dysfunction	18.584 (6.918–49.921)	<.001		
History of CHD	5.206 (2.267–11.953)	<.001		
Dyslipidemia	1.059 (0.522–2.149)	.874		
Total ischemic time	1.016 (1.011–1.021)	<.001	1.007 (1.001–1.013)	.015
LAD	10.191 (4.067–25.534)	<.001	4.030 (1.208–13.443)	.023
LCX	0.321 (0.094–0.1.096)	.070		
RCA	0.105 (0.031–0.351)	<.001		
Multivessel disease	11.337 (4.995–25.731)	<.001		
CA125 ≥ 13.2 U/mL	17.696 (6.977–44.879)	<.001	5.113 (1.561–16.753)	.007
NT-proBNP ≥ 2300 pg/mL	21.561 (9.305–49.958)	<.001	3.257 (1.065–9.962)	.038

Data are represented as OR (95% CI), *P* value.

CA125 = carbohydrate antigen 125, CHD = coronary heart disease, DBP = diastolic blood pressure, HR = heart rate, LAD = Left anterior descending branch, LCX = Left circumflex branch, NT-proBNP = N-terminal pro-B-type natriuretic peptide, RCA = right coronary artery, SBP = systolic blood pressure.

### 3.4. Odds ratios of CA125 combined with NT-proBNP to AHF

Based on CA125 and NT-proBNP optimal cutoff value, the patients were divided into 3 groups (C1, C2, C3): 2 cases (1.45%) in C1 group, 12 cases (20.34%) in C2 group, and 25 cases (69.44%) in C3 group with AHF. Logistic regression model analysis: the relative odds ratio of C1 was set as 1.00, univariate logistic regression analysis found that compared with C1, C2 (OR = 17.36, 95% CI: 3.75–80.44, *P* < .001), C3 (OR = 154.55, 95% CI: 32.29–739.76, *P* < .001). After adjusting for clinical confounding variables: compared with C1, C2 (OR = 6.41, 95% CI: 1.22–33.84, *P* = .029), C3 (OR = 19.27, 95% CI: 3.12–118.92, *P* = .001) (Table 4).

**Table 4:** The odds ratios of CA125 combined with NT-proBNP subgroup for acute heart failure.

Group	Unadjusted odds ratio		Adjusted odds ratio	
C1	1.000		1.000	
C2	17.362 (3.747–80.440)	<.001	6.410 (1.215–33.835)	.029
C3	154.545 (32.286–739.762)	<.001	19.265 (3.121–118.921)	.001

Data are represented as OR (95% CI), *P* value.

CA125 = carbohydrate antigen 125, NT-proBNP = N-terminal pro-B-type natriuretic peptide.

C1 = (CA125 < 13.20 U/mL and NT-proBNP < 2300 pg/mL, n = 138).

C2 = (CA125 ≥ 13.20 U/mL or NT-proBNP ≥ 2300 pg/mL, n = 59).

C3 = (CA125 ≥ 13.20 U/mL and NT-proBNP ≥ 2300 pg/mL, n = 36).

## 4. Discussion

This study mainly found that CA125 and NT-proBNP were positively correlated with Killip grading after PPCI in STEMI patients, and the combination of CA125 and NT-proBNP could improve the predictive efficiency of developing AHF during hospitalization.

In the era of PPCI, the incidence of AHF in STEMI decreased, but the prognosis remained severe once heart failure occurred.^[5]^ In 1967, Killip and Kimball first proposed the stratification of cardiac function in STEMI patients based on physical examination, which has long been used to assess the severity of cardiac function in the acute phase following acute myocardial infarction and to predict short - and long-term mortality.^[2,3,16]^ Nevertheless, in some cases, the symptoms and signs of congestion are not typical and cannot be easily recognized early.^[17]^ In consequence, more accurate indicators of congestion identification are needed to guide clinical practice.

For the past few years, due to a deeper understanding of different biomarkers, it has become one of the research directions of precision medicine to try to improve the risk identification of patients with acute myocardial infarction by combining different biomarkers.^[18]^ Natririuretic peptides are released by the ventricle in response to volume overload and mechanical stress stretch, and have been shown to be valuable in the prediction of heart failure and adverse events after STEMI.^[19]^ CA125, a kind of high molecular glycoprotein, has been considered as a potential new biomarker related to heart failure. It is believed that hyperemia, serosal effusion and inflammatory stimulation can promote its release, and the mechanism may be related to the response of serosal mesothelial cells to serosal effusion and/or proinflammatory stimulation.^[9]^ When STEMI and AHF merge together, it is a complex pathophysiological process of congestion accompanied by an underlying inflammatory cascade.^[12]^ De Gennaro et al^[20]^ found that CA125 was more specific (97.1 vs 31.4%) and accurate (83.0 vs 48.9%) than BNP in the identification of pulmonary congestion, and the combination of these two biomarkers further improved the identification efficiency. However, the study did not distinguish the types of myocardial infarction and only 47 patients were included. Thus, the hypothesis presented in this study is that a combination of biomarker strategies with different pathophysiological mechanisms may provide a more accurate risk assessment of AHF after STEMI.

Analysis of data from 233 STEMI patients undergoing PPCI showed that the incidence of AHF during hospitalization was 16.74% (39/233), and both CA125 and NT-proBNP were positively associated with killip classifications. According to ROC curves analysis (Fig. 1), the optimal cutoff points of CA125 and NT-proBNP for AHF recognition were 13.20 U/mL and 2300 pg/mL, respectively, with sensitivities of 84.62% and 74.36% and specificity of 81.44% and 89.18%. The AUC of CA125 combined with NT-proBNP was higher than that of CA125 or NT-proBNP (DeLong test, *P* < .05) (Fig. 2), indicating that the combination of these two biomarkers can improve the recognition efficiency. The above findings were similar to those of Falcao et al,^[21]^ who found that the optimal cutoff value for CA125 and NT-proBNP to predict pulmonary congestion in STEMI patients was 12.45 U/mL and 2010 pg/mL. However, the higher cut point for diagnosis AHF in this study may be related to the ethnicity and age of the enrolled patients. In addition, multiple logistic regression analysis of clinical variables showed that age, culprit vessel of LAD, total ischemic time, CA125, and NT-proBNP were independent predictors of AHF during hospitalization. These results were consistent with previous reports that age, anterior wall myocardial infarction, prolonged ischemia time, and NT-proBNP are predictors of AHF following STEMI.^[14,22]^ Interestingly, the new findings in this study indicate that CA125 is also an independent predictor of AHF, suggesting that vigilance should be increased for subsequent AHF in STEMI patients with advanced age, longer total ischemia time, culprit vessel of LAD, elevated CA125 or/and NT-proBNP, and that timely intensive therapy is necessary.

Furthermore, based on CA125 and NT-proBNP truncation values, all subjects were divided into three groups for analysis: taking C1 group as a reference (setting OR = 1.00), C2 (OR = 6.41, 95% CI: 1.22–33.84, *P* = .029), C3 (OR = 19.27, 95% CI: 3.12–118.92, *P* = .001) (Table 4). This trend indicates that compared with C1 group, the relative risk of developing AHF was 6.410 times when either CA125 or NT-proBNP was above the cutoff value (C2), while the relative risk of AHF was 19.265 times when both of them were higher (C3), reaching the maximum.

In conclusion, CA125 and NT-proBNP were positively correlated with Killip classifications in STEMI patients treated with PPCI, both of which were independent predictors of the occurrence and development of AHF. CA125 combined with NT-proBNP can improve the predictive efficiency of AHF during hospitalization. Because of its long half-life, easy detection and low price, CA125 is worthy of rational clinical application. Limitations of this study: This study was a single-center observational study with a small sample size, which affects the accuracy of the results. Due to data limitations, the value of CA125 changes in post-discharge follow-up and its influence on long-term prognosis have not been observed, which may be one of the directions of future research.

## Acknowledgments

The authors thank all the participants who volunteered to participate in our study.

## Author contributions

**Conceptualization:** Meifang Wu.

**Data curation:** Kaizu Xu, Xi Chen.

**Formal analysis:** Kaizu Xu, Meinv Huang, Xi Chen.

**Funding acquisition:** Meifang Wu, Xi Chen.

**Investigation:** Kaizu Xu, Meinv Huang, Xiuping Zhuo, Yujuan Weng.

**Methodology:** Xi Chen.

**Project administration:** Meifang Wu, Xi Chen.

**Resources:** Meifang Wu, Xiuping Zhuo, Yujuan Weng.

**Software:** Kaizu Xu.

**Supervision:** Meifang Wu.

**Writing – original draft:** Kaizu Xu.

**Writing – review & editing:** Xi Chen.
